# Recurrent Tuberculosis and Exogenous Reinfection, Shanghai, China

**DOI:** 10.3201/eid1211.051207

**Published:** 2006-11

**Authors:** Guomiao Shen, Zhen Xue, Xin Shen, Bin Sun, Xiaohong Gui, Mei Shen, Jian Mei, Qian Gao

**Affiliations:** * Fudan University, Shanghai, People's Republic of China;; † Shanghai Municipal Centers for Disease Control and Prevention, Shanghai, People's Republic of China

**Keywords:** *Mycobacterium tuberculosis*, tuberculosis, recurrence, molecular epidemiology, DNA fingerprinting, dispatch

## Abstract

Of 52 patients with recurrent tuberculosis in Shanghai, People's Republic of China, 32 (61.5%) had isolates in which genotype patterns of *Mycobacterium tuberculosis* differed between first and second episodes. This result indicates that exogenous reinfection is common in an area with a high incidence of tuberculosis.

Elucidating the role of reinfection in tuberculosis (TB) recurrence is important in the People's Republic of China because this country has the second highest incidence of TB in the world, an estimated rate in 2004 of 101 cases/100,000 persons/year (*1*). After effective short-course therapy for active TB, some patients experience another, recurrent TB episode. The recurrent episode may be due to endogenous reactivation or exogenous reinfection. The role of exogenous reinfection has been debated for decades (*2**,**3*). Understanding the cause for recurrence helps clinicians evaluate the effectiveness of therapeutic regimens and TB prevention and control programs to assess strategies and interventions.

DNA fingerprinting techniques provide excellent tools to address whether recurrent TB is caused by endogenous reactivation or exogenous reinfection. Different Mycobacterium tuberculosis strains can be differentiated by genotyping methods that use information about genetic markers and their distribution in the genome (*4*). Among persons with recurrent TB, if the isolates from 2 TB episodes have the same genotype, the episode is defined as an endogenous relapse; otherwise, it is defined as exogenous reinfection. Previously, researchers have tried to assess the relative importance of endogenous relapse versus exogenous reinfection, with varied results (*2**,**5**–**10*). Our study helps elucidate the role of reinfection in TB recurrence in China.

## The Study

Shanghai is 1 area in China with high TB treatment success rates. Persons with TB symptoms (mainly cough for at least 2 weeks, chest pain, weight loss, and fever) can go to any hospital or community health center in Shanghai. They are first screened by chest radiograph. All patients with suspected TB are referred to a TB hospital, where sputum is examined by smear and culture. TB is bacteriologically confirmed if >1 sputum smear examination result was positive for acid-fast bacilli or if the culture was positive. The TB hospital sends all mycobacteria-positive cultures to the TB reference laboratory at the Shanghai Municipal Centers for Disease Control and Prevention (Shanghai CDC), which participated in the World Health Organization/International Union against Tuberculosis and Lung Disease global drug resistance surveillance project, for species identification and drug susceptibility testing. TB patients are treated in the TB hospital during the intensive phase. On the basis of the sputum smear and culture examination 1 or 2 months after TB therapy is initiated, the patient is discharged from the hospital and finishes treatment as an outpatient. The community health center physician trains family members to supervise and observe the TB patient's remaining doses and treatment. Completion of anti-TB therapy is based on the examination of sputum smear, culture, and chest radiographic results. Shanghai CDC collects and manages patient information, such as social and demographic characteristics, clinical data, TB treatment regimens, and the result of drug susceptibility testing and species identification.

**Figure 1:**
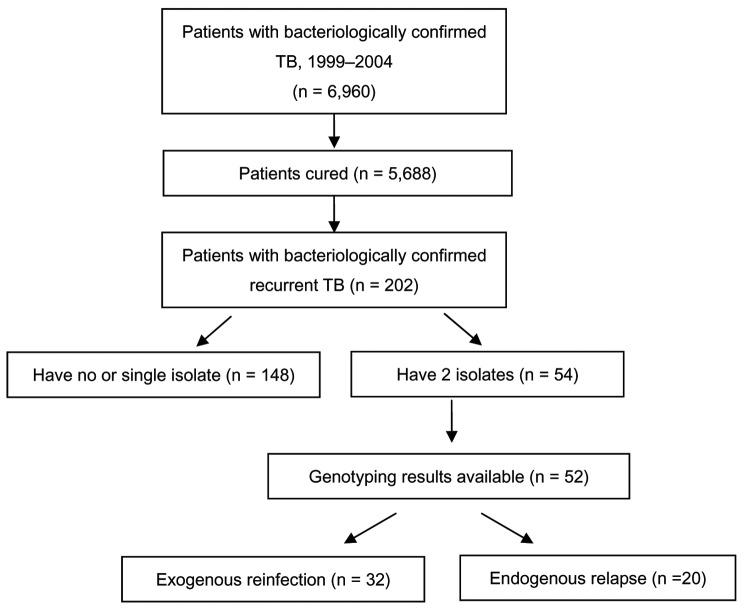
Selection of patients in the study, Shanghai, People's Republic of China, 1999–2004. TB, tuberculosis. The total number of patients from 1999 through 2004 was 6,960; among these patients, 5,688 were cured.

From January 1999 through September 2004, Shanghai CDC collected 6,442 clinical isolates from a total of 6,960 persons with bacteriologically confirmed (by smear or culture) TB. Of these case-patients, 5,688 were cured, and 202 (164 male and 38 female) had a recurrence, defined by the following criteria: 1) their TB episode was confirmed by culture; and 2) they experienced 2 successive TB episodes, with cure as the outcome of the first episode. Cure was defined as the completion of a standard course of combination therapy and successive negative sputum cultures during treatment. At the same time, chest radiography showed resolution of the focus of infection. On the basis of the selection criteria, 54 patients with recurrent TB were included in the study (Figure 1).

**Figure 2:**
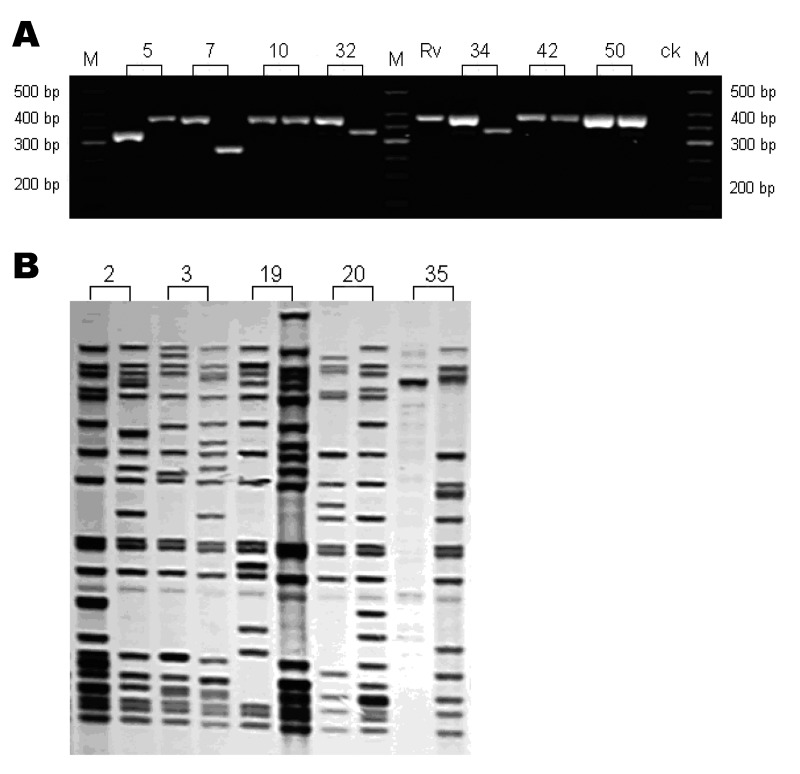
Genotyping analysis of clinical isolates from patients with recurrent tuberculosis. Numbers represented the patients' codes. A) Gel electrophoresis analysis of the PCR products of the mycobacterial interspersed repetitive unit (MIRU) locus 10. bp, base pair; M: DNA marker; Rv, H37Rv positive control; ck, negative control. B) IS*6110* restriction fragment length polymorphism analysis of some patients with different MIRU patterns.

The mycobacterial interspersed repetitive unit (MIRU) typing method (*11*) was used to genotype strains from these patients. This method is relatively easier to perform and less technically demanding than IS6110 restriction fragment length polymorphism (IS6110-RFLP), which was used in many previous molecular epidemiologic studies of TB (*12*). We followed the protocol described by Kwara et al. (*13*) with modifications. PCR products were analyzed by 2.5% (w/v) agarose gel electrophoresis (Figure 2A). Genotyping was performed for 2 isolates. We analyzed the data for the remaining 52 patients and found MIRU patterns for both episodes to be the same for 20 patients and different for 32. Of these 32 patients, 13 had 1 MIRU locus change between the 2 isolates, 10 had a change in 2 loci, and 9 had >3 loci changes in their isolates (Table A1). These results indicate that 32 (61.5%) of 52 (95% confidence interval 47.0%–74.4%) of the recurrent cases were due to reinfection. To further validate the MIRU genotype result, the IS6110 RFLP genotyping method was performed; results showed that the isolates with 1 or 2 MIRU locus changes had very different RFLP patterns (a difference in >4 bands in the IS6110-RFLP, Figure 2B).

We further used patient age group and intervals between the 2 episodes to classify recurrent TB. We found that the percentage of TB patients with an exogenous reinfection decreased with age from 100% (TB patients <30 years of age) to 66.7% (TB patients 30–60 years) and 53.3% (TB patients >60 years). We also found that the frequency of exogenous reinfection increased with the amount of time that elapsed between the end of TB treatment for the first episode of TB and the date that the second episode was diagnosed. Exogenous reinfection accounted for 7 (46.7%) of the 15 recurrent episodes that occurred within 6 months after treatment for the first episode; the percentage of recurrent cases due to exogenous reinfection increased to 73.9% (17/23) among TB patients whose second episode occurred >1 year after treatment for the first episode.

## Conclusions

We analyzed genotypes of 104 isolates from 52 patients who experienced 2 TB episodes from 1999 through 2004. Thirty-two of 52 patients had different MIRU genotype patterns in clinical isolates from their 2 episodes, which indicates that exogenous reinfection accounted for 61.5% of the recurrent cases in Shanghai during the study period. The high proportion of exogenous reinfection in recurrent TB patients indicates that high levels of transmission of M. tuberculosis are an important cause of TB in Shanghai, China.

Although several reports have indicated that exogenous reinfection may occur after successful treatment, the proportion of TB cases that are actually caused by exogenous reinfection may vary dramatically for several reasons, such as the patients' HIV infection status, different facilities, and healthcare providers' various definitions (some used different numbers of days elapsed between the first and second episode to define a recurrent case of TB), and particularly the small sample size (*2*). Several studies have reported that HIV may be a risk factor for exogenous reinfection (*2**,**14**,**15*). Unfortunately, we do not have data on each patient's HIV infection status, and we cannot totally exclude the effect of HIV infection. However, considering the low incidence of HIV infection among residents of Shanghai (≈0.6 cases/100,000 persons/year), we consider it unlikely that HIV is a major factor in our findings. The criteria used to define recurrent TB cases differ; various studies defined the interval between the end of TB treatment and a new episode (recurrent TB) as 3–12 months (*5**,**7**,**8*). Our study did not define the interval and determined that 61.5% of the recurrent TB cases were due to exogenous reinfection. If we chose a 6-month interval to define a recurrent TB case, then we would find an even higher percentage of recurrent TB cases were due to exogenous reinfection (67.6%, 25/37).

Previous studies and our study demonstrate that TB patients can be reinfected with a new strain of M. tuberculosis after treatment, which indicates that the immunity evoked by the primary infection does not protect the patient against a later infection. A recent study from South Africa demonstrated that the rate of TB reinfection after successful treatment is even higher than the rate of new TB infection (*5*). Such results suggest major challenges for the development of a new vaccine that will be effective against M. tuberculosis.

In summary, our study showed that 61.5% of recurrent TB cases in Shanghai from 1999 through 2004 were due to exogenous reinfection and confirmed that reinfection may be common in areas with a relatively high incidence of TB. This finding provides important implications for TB control. To prevent recurrent TB, more attention should be paid to the interruption of TB transmission.

## Appendix

**Table A1:** Characteristics of 52 patients with recurrent tuberculosis*

Patient no.	Sex	Age (y)	Relapse time (mo)	MIRU genotype		
				First episode	Second episode	Changed no. of loci
1	M	80	4	223325163523	222325163533	2
2	M	84	20	243325163533	233325163532	2
3	M	44	29	222325163533	223325153532	3
4	M	60	5	223325163833	223325163833	0
5	M	42	13	222325163533	223325163433	2
6	M	75	20	223325163633	223325163631	1
7	M	41	10	223325173533	221325173533	1
8	M	61	49	223325163533	223325163533	0
9	M	77	3	223325163533	223325153433	2
10	M	41	5	223325173531	223325163533	2
11	M	74	11	222325163533	222325163533	0
12	M	77	5	223325163533	223325163533	0
13	M	46	8	242325252322	252325152322	2
14	M	40	3	223325143534	223325143534	0
15	M	61	14	222225132322	223325163533	7
16	M	56	1	223325163533	223325163533	0
17	M	65	15	223325163533	223325163533	0
18	M	55	11	223315163533	223315163533	0
19	F	68	26	223325163533	222325163533	1
20	M	69	31	223325163533	223425163533	1
21	M	69	10	223325173533	223325173533	0
22	M	68	16	223325163434	221325183533	4
23	M	74	42	223225183533	223325163533	2
24	M	67	39	222325173533	223325163533	2
25	M	87	16	223325163533	223325163533	0
26	M	41	40	223425163533	223225163533	1
27	M	80	10	223325143533	223325143533	0
28	M	86	17	223325163533	223325153533	1
29	M	77	23	223425173533	223425173533	0
30	M	49	18	223325163533	223325173533	1
31	M	69	6	223325163633	223325163633	0
32	M	62	7	223325163533	222325163533	1
33	M	26	14	222225143423	222325142322	4
34	M	50	9	223325173533	142325134323	7
35	F	27	33	223425173563	222225152323	6
36	M	60	6	223325173533	222325173533	1
37	M	47	7	223325153433	223325163533	2
38	M	52	4	223325153533	223325173533	1
39	M	74	7	223325163533	223325163533	0
40	M	68	2	223325173533	223325163533	1
41	M	28	8	213225163533	223325173533	3
42	M	70	30	223325163533	223325153523	2
43	M	72	2	223325163633	223325163633	0
44	M	57	51	223325163533	223325163533	0
45	M	46	28	223325163533	223325163533	0
46	M	73	1	222325173543	222325153532	3
47	M	45	1	223325163533	223325163533	0
48	M	46	10	222325163534	222325263534	1
49	M	84	8	223325153433	223325153433	0
50	F	42	9	223325163433	223325163533	1
51	M	15	13	223325173533	252425144332	7
52	M	67	5	223325163533	223325163533	0

*M, male; F, female. Each digit of the mycobacterial interspersed repetitive unit (MIRU) genotype represents a locus. The 12 MIRU loci are 2, 4, 10, 16, 20, 23, 24, 26, 27, 31, 39, and 40.

## References

[1] World Health Organization. Global tuberculosis control: surveillance, planning, financing. Geneva: The Organization; 2006. (WHO/HTM/TB/.362).

[2] Lambert ML, Hasker E, van Deun A, Roberfroid D, Boelaert M, van der Stuyft P. Recurrence in tuberculosis: relapse or reinfection? Lancet Infect Dis. 2003;3:282–7. 10.1016/S1473-3099(03)00607-8 12726976

[3] Chiang CY, Riley LW. Exogenous reinfection in tuberculosis. Lancet Infect Dis. 2005;5:629–36. 10.1016/S1473-3099(05)70240-1 16183517

[4] Barnes PF, Cave MD. Molecular epidemiology of tuberculosis. N Engl J Med. 2003;349:1149–56. 10.1056/NEJMra021964 13679530

[5] Verver S, Warren RM, Beyers N, Richardson M, van der Spuy GD, Borgdorff MW, Rate of reinfection tuberculosis after successful treatment is higher than rate of new tuberculosis. Am J Respir Crit Care Med. 2005;171:1430–5. 10.1164/rccm.200409-1200OC 15831840

[6] van Rie A, Warren R, Richardson M, Victor TC, Gie RP, Enarson DA, Exogenous reinfection as a cause of recurrent tuberculosis after curative treatment. N Engl J Med. 1999;341:1174–9. 10.1056/NEJM199910143411602 10519895

[7] Bandera A, Gori A, Catozzi L, Degli Esposti A, Marchetti G, Molteni C, Molecular epidemiology study of exogenous reinfection in an area with a low incidence of tuberculosis. J Clin Microbiol. 2001;39:2213–8. 10.1128/JCM.39.6.2213-2218.2001 11376059 PMC88113

[8] Caminero JA, Pena MJ, Campos-Herrero MI, Rodriguez JC, Afonso O, Martin C, Exogenous reinfection with tuberculosis on a European island with a moderate incidence of disease. Am J Respir Crit Care Med. 2001;163:717–20. 11254530 10.1164/ajrccm.163.3.2003070

[9] Warren RM, Streicher EM, Charalambous S, Churchyard G, van der Spuy GD, Grant AD, Use of spoligotyping for accurate classification of recurrent tuberculosis. J Clin Microbiol. 2002;40:3851–3. 10.1128/JCM.40.10.3851-3853.2002 12354898 PMC130897

[10] Jasmer RM, Bozeman L, Schwartzman K, Cave MD, Saukkonen JJ, Metchock B, Recurrent tuberculosis in the United States and Canada: relapse or reinfection? Am J Respir Crit Care Med. 2004;170:1360–6. 10.1164/rccm.200408-1081OC 15477492

[11] Supply P, Lesjean S, Savine E, Kremer K, van Soolingen D, Locht C. Automated high-throughput genotyping for study of global epidemiology of Mycobacterium tuberculosis based on mycobacterial interspersed repetitive units. J Clin Microbiol. 2001;39:3563–71. 10.1128/JCM.39.10.3563-3571.2001 11574573 PMC88389

[12] Scott AN, Menzies D, Tannenbaum TN, Thibert L, Kozak R, Joseph L, Sensitivities and specificities of spoligotyping and mycobacterial interspersed repetitive unit-variable-number tandem repeat typing methods for studying molecular epidemiology of tuberculosis. J Clin Microbiol. 2005;43:89–94. 10.1128/JCM.43.1.89-94.2005 15634955 PMC540143

[13] Kwara A, Schiro R, Cowan LS, Hyslop NE, Wiser MF, Roahen Harrison S, Evaluation of the epidemiologic utility of secondary typing methods for differentiation of Mycobacterium tuberculosis isolates. J Clin Microbiol. 2003;41:2683–5. 10.1128/JCM.41.6.2683-2685.2003 12791904 PMC156564

[14] Godfrey-Faussett P, Githui W, Batchelor B, Brindle R, Paul J, Hawken M, Recurrence of HIV-related tuberculosis in an endemic area may be due to relapse or reinfection. Tuber Lung Dis. 1994;75:199–202. 10.1016/0962-8479(94)90008-6 7919312

[15] Sonnenberg P, Murray J, Glynn JR, Shearer S, Kambashi B, Godfrey-Faussett P. HIV-1 and recurrence, relapse, and reinfection of tuberculosis after cure: a cohort study in South African mine workers. Lancet. 2001;358:1687–93. 10.1016/S0140-6736(01)06712-5 11728545

